# The Association between Combined Lifestyle Factors and All-Cause and Cause-Specific Mortality in Shiga Prefecture, Japan

**DOI:** 10.3390/nu12092520

**Published:** 2020-08-20

**Authors:** Sae Tanaka, Aya Kito, Eri Imai

**Affiliations:** Department of Nutrition, School of Human Cultures, The University of Shiga Prefecture, 522-8533 Hikone City, Japan; oi34stanaka@ec.usp.ac.jp (S.T.); oi34akito@ec.usp.ac.jp (A.K.)

**Keywords:** mortality, standard mortality ratio, diet, lifestyle, life expectancy, cancer, heart diseases, cerebrovascular diseases

## Abstract

Background: To decrease mortality, the benefit of combined healthy lifestyles has been suggested but is still unclear, especially for cause-specific mortality. We examined the relationship between combined lifestyle factors and all-cause and cause-specific mortality in Shiga prefecture, Japan. Methods: This was an ecological study of 19 municipalities, using the data from the 2008–2012 standard mortality ratio (SMR) reported by the Ministry of Health and Welfare and the 2015 Health and Nutrition Survey in Shiga prefecture. The health behaviors score was calculated based on five factors (ranging from 0 to 5): diet quality (assessed adherence to dietary reference intake for Japanese), smoking, alcohol drinking, regular exercise, and sleep duration. In the multiple linear regression, the relationships between the health behaviors score and SMR of all-cause, cancer, heart diseases, and cerebrovascular diseases were estimated by sex. Results: The health behaviors score was negatively associated with the cancer SMR in women (*β* = −0.968, *p* = 0.011). For other causes, no significant association was found for either sex. A greater proportion of those who never smoked (*β* = −0.780, *p* = 0.016) and those who had a higher quality diet (*β* = −0.703, *p* = 0.048) were associated with lower cancer SMR in women. Women’s intake of some micronutrients, particularly fruits, was higher than men. This study suggests that a combination of health behaviors, especially never smoking and high-quality diet intake are associated with lower cancer SMR in women and could be helpful in prolonging life expectancy.

## 1. Introduction

Japan is one of the countries with the greatest longevity in the world [1]. According to the Japanese Ministry of Health and Welfare, in 2015, the Shiga prefecture had the highest life expectancy in Japan for men, at 81.8 years, and the fourth highest for women, at 87.6 years [2]. The trend of life expectancy in Shiga prefecture from 1965 to 2015 increased more than that of the whole country [3]. Thus, Shiga prefecture has recently been receiving a lot of attention as the longevity prefecture.

There are reports that in Japan, increased life expectancy is due to reduced mortality from cardiovascular diseases (CVD) and neoplasms [4]. In the Shiga prefecture, the age-adjusted mortality ratio of cerebrovascular diseases and cancer were decreased by years, and the standard mortality ratio (SMR) of those causes was lower than in other Japanese prefectures [5].

Lifestyle factors have been shown to be independently related to the risks of all-cause and cause-specific mortality. Meta-analyses have reported that current smoking is a risk for all-cause and lung cancer death [6], and a higher intake of fruits and vegetables reduces risk of all-cause, CVD, and cancer mortality [7]. A large prospective cohort study has shown that physical activity, which is a moderate to vigorous physical activity in leisure, reduces the risk of all-cause mortality and CVD [8].

Recently, the synergetic effect of combined lifestyle factors has been investigated [9]. A prospective cohort study of Italian adults has revealed that combining the Mediterranean diet, nonsmoking, and physical activity is associated with a lower risk of all-cause mortality compared with each of those factors independently [10]. Given that people have more concurrent lifestyle factors, more lifestyle factors should be considered. Although some studies have assessed more than four lifestyle factors, the factors tended to include nonbehavioral factors, such as body mass index (BMI) and waist circumference [11,12]. The studies that have investigated the association between combined lifestyle factors and cause-specific mortality are fewer than those that have assessed all-cause mortality. CVD and cancer are two of the major causes of death both nationally and globally [4,13]; thus, investigating these associations might reveal some reasons for extended life expectancy. We eventually sought to clarify the characteristics of longevity in the prefectures, which would be useful for targeted public health efforts nationally and globally. This study aimed to examine the relationship between multiple lifestyle factors and all-cause and cause-specific mortality in Shiga prefecture, Japan. We hypothesized that the higher the number of healthy lifestyle factors, the lower mortality from all-cause, CVD, and cancer.

## 2. Materials and Methods

### 2.1. Study Design

This study was an ecological observational study. Shiga prefecture, Japan, is comprised of 13 cities and 6 towns.

### 2.2. Mortality Data

The total number of deaths and SMR at the municipality level during the period 2008–2012 were obtained from the specified report of vital statistics of the Ministry of Health and Welfare [5]. This official report contains multifaceted information about births, deaths, stillbirths, marriages, and divorces based on vital statistics. From the report, we used the most recent data on death at the public health center and municipality level. The SMR was calculated by dividing the observed number of deaths in each municipality by an expected number of deaths in each municipality using the following formula.

(1) $$\mathrm{SMRr}=\frac{\sum_{j}d_{jr}}{\sum_{i}t_{ir}\left(\frac{\sum jD_{ij}}{T_{i}\times 5}\right)\times 5}\times 100$$

where *T_i_* = age-specific population of the standard population in 2010, *D_ij_* = year- and age-specific number of deaths of standard population, *t_ir_* = age-specific population of each municipality in 2010, and *d_jr_* = year-specific number of deaths of each municipality. *r* = municipality, *i* = 5-year age group, and *j* = year. The causes of death that we investigated and their association with lifestyle factors were heart diseases, cerebrovascular diseases, and cancer because these causes were the top three causes of death in Shiga prefecture in 2017 [14].

### 2.3. Dietary and Lifestyle Data

Individual level data were obtained from the Health and Nutrition Survey of Shiga prefecture (HNSS), Japan, in 2015. The HNSS is a survey that covers approximately 4000 households, once every 5 years, in randomly selected census units defined by the Shiga prefecture. It provides the largest prefecturally representative sample available by which to monitor dietary intake and lifestyle factors of the people in the Shiga prefecture. The most recent data in 2015, covering 4229 households chosen randomly from approximately 1% of the households of all 19 cities in Shiga, involved all household members aged ≥1 year. The HNSS was conducted on one day in November 2015. The nutrient and food intake were estimated by a 1-day household dietary record, including the approximate proportions in which each dish was divided among the family members. Information on lifestyle and subjective health was obtained from a self-administered questionnaire. BMI was calculated as body weight (kg) divided by height squared (m^2^).

In the present study, from a total of 11,281 individuals participated to HNSS in 2015, 1663 individuals aged <20 years were excluded. In addition, a total of 2398, 240, 358, and 247 individuals who provided incomplete data for the survey on diet, health status, BMI, and lifestyle factors (smoking, exercise, sleeping, and alcohol drinking), respectively, were also excluded. Then, 318 individuals with an implausibly high or low energy intake (in the upper or lower 2.5% of the distribution of energy intake, respectively) were excluded from the study. Ultimately, data from 6057 individuals (2790 men and 3267 women) remained and were divided into 13 cities and 6 towns by individual’s municipality code.

#### 2.3.1. Ethics Statement

The HNSS, conducted by the government of Shiga prefecture, has stringent protocols and procedures that ensure confidentiality and protect individual participants from being identified. The present analysis was based on a secondary analysis of observational survey data. The present study was approved by the Shiga prefecture in Japan. The Shiga prefecture government anonymized all individual-level data before providing the authors with the datasets for this study. No ethical review was sought based on the Ethical Guidelines for Medical and Health Research Involving Human Subjects, given this study used only information that had already been anonymized.

#### 2.3.2. Availability of Data and Materials

The datasets analyzed during the current study are not publicly available due to a secondary analysis of the observational survey but are available from the corresponding author upon reasonable request.

### 2.4. Health Behaviors Score

#### 2.4.1. Diet Quality

We established the health behaviors score referring to previous studies [15,16,17]. The nutrient and food intakes of each household were estimated using a 1–day dietary record. The quality of the diet was assessed with the reference values given in the Dietary Reference Intakes for Japanese 2015 (DRIs-J) [18] modified from the previously developed score [19,20]. In this study, the adequacy of the 18 nutrients intakes assessed included protein, fat, carbohydrate, vitamin A, vitamin B_1_, vitamin B_2_, niacin, vitamin B_6_, vitamin B_12_, folic acid, vitamin C, calcium, magnesium, iron, zinc, copper, dietary fiber, and saturated fatty acid using the recommended daily allowance (RDA) and tentative dietary goal (DG). In the DRIs-J 2015, the adequate intake (AI) was set for some nutrients for cases in which the RDA could not be set due to insufficient scientific evidence. Because the AI was developed using the median of intakes in population, even if the nutrient intake of individuals is below the AI, it is insufficient to use it for evaluation. Thus, this study excluded these nutrients from the evaluation targets. For the 18 nutrients included in this study, fat, carbohydrate, dietary fiber, and saturated fat were given the DG as reference values. Sodium was also given the DG in the DRIs-J 2015. However, it was not assessed in this study because the value was set higher than the one derived from scientific evidence for preventing or managing lifestyle-related diseases due to the current Japanese status of sodium intakes. For each of the 18 nutrients, a score of 1 was allocated if the nutrient met or exceeded the RDA, and 0 if it did not meet the RDA. Additionally, the nutrient having a tolerable upper intake level (UL) was given a score of 1 if the nutrient was below UL, and 0 if it was above the UL. Energy intake was assessed using the estimated energy requirement (EER). EER = basal metabolic reference value (kcal/kg body weight/day) × body weight (kg) × physical activity level. Basal metabolic reference values were specific for sex and age. A score of 1 was allocated if an individual’s energy intake was ≥EER with physical activity level (PAL) = 1 and ≤EER with PAL = 3, and 0 if it deviated from the range. The range of PAL values were 1.50–2.00 for participants aged 20–69 years and 1.45–1.95 for participants aged ≥70 years, in this study. In the DRIs−J 2015, PALs calculated from the measured energy expenditure and estimated basal metabolic rate of healthy Japanese adults [21] were used. PALs of 1, 2, and 3 indicated as low, moderate, and high corresponded to a sedentary lifestyle, sedentary work, which includes housework and light intensity sports, and work with high-intensity physical activity or leisure-time physical activity, respectively. The total DRIs-J score was constructed by summing the scores of the 18 nutrients and energy intake. The DRIs-J scores ranged from 0 to 19. Each participant was given 1 point for a DRIs-J score greater than or equal to the median score by sex in the participants. The median score was 7.0 for both sexes while the mean score was 7.8 ± 4.2 for men and 8.1 ± 4.4 for women.

#### 2.4.2. Lifestyle Factors

Information on height, body weight, smoking, exercise, sleeping, alcohol drinking, and more was obtained from the self-administered questionnaire. Smoking was assessed with three options: current or former smoker or never smoked. Participants who never smoked were given 1 point. Current and former smokers were scored 0 to avoid misclassification for those who stopped smoking recently. Information on alcohol drinking was obtained regarding the frequency and quantity by using 2 questions. The frequency of alcohol drinking was reported with seven options: “never”, “past”, “occasionally”, “1–2 times per week”, “3–4 times per week”, “5–6 times per week”, and “every day”. If participants answered “never”, “past”, or “occasionally” for the first question, they were scored 1. If participants consumed alcoholic beverages 1–2 times per week or more, a second question was asked about the average quantity consumed per day. Alcohol consumption ≥40 g/d for men or ≥20 g/d for women was scored 0. Alcohol consumption <40 g/d for men or <20 g/d for women was scored 1. Excessive amounts of alcohol have been reported to be associated with a higher risk of lifestyle-related diseases [22,23,24], and alcohol consumption <40 g/d for men and <20 g/d for women has been officially recommended in Japan [25].

Exercise was assessed with 2 questions. First, individuals were asked “How often do you regularly do physical activity consciously for your health?” Participants chose from 4 options: “never”, “past”, “sometimes”, and “usually”. If participants answered “never” or “past” for the first question, they were scored 0.

Those who answered “sometimes” and “usually” then answered the following question: “Have you done 30 min of exercise at least twice a week for a year?” The answer consisted of 3 options: “past”, “never”, and “current”. Those who answered “sometimes” or “usually” for the first question and “current” for the second question were given 1 point, whereas those who answered “sometimes” or “usually” for the first question and “never” or “past” for the second question were scored 0. The amount of exercise equal to 4 metabolic equivalent (METs) hours per week has been reported to be associated with a low risk of all-cause and cancer mortality [11,26] and is recommended in the Japanese national health promotion: Health Japan 21 (the second term) [25].

Sleep duration was assessed using the question, “How many hours do you usually sleep per day?” The response categories were: <5 h/d, ≥5 h/d and <6 h/d, ≥6 h/d and <7 h/d, ≥7 h/d and <8 h/d, ≥8 h/d and <9 h/d, and ≥9 h/d. Those who answered ≥7 h/d and <8 h/d or ≥8 h/d and <9 h/d were given 1 point. The rest were scored 0. This duration has been suggested to have an association with a low risk of all-cause mortality [27].

Table 1 shows the scoring of health behaviors in the study. The health behaviors score was estimated based on whether individuals met each of the 5 criteria: diet quality, smoking, alcohol drinking, exercise behavior, and sleeping. The health behaviors score ranged from 0 to 5, with a higher score indicating a higher number of good lifestyle behaviors.

### 2.5. Statistical Analysis

Diet, lifestyle, and other participant’s characteristics were calculated to the mean value or proportion of the 19 municipalities. Multiple linear regression was used to investigate the association between SMR and the health behaviors score, and the 5 lifestyle factors consisted of the health behaviors score. Although there were differences between the SMR survey years and lifestyle factors, it has been reported that the ranking of leading causes of death in Japan did not change between 2005 and 2015 [4]. Spearman’s correlation coefficient was used to evaluate the relationships between SMR and potential confounders: age, BMI, energy intake, and subjective health. These were selected on plausibility and the literature. Age, BMI, and energy intake were treated as continuous variables, whereas subjective health was a binary variable (Excellent, Good or Average/Poor or Very Poor). Then, variables with a *p*-value of <0.10 were considered for inclusion for adjustment in the multiple linear regression models. These putative variables were specific to sex and cause: BMI for all causes in men and women; age and energy intake for cancer in men; age and BMI for cancer in women; heart diseases in men, and cerebrovascular diseases in women. In the multiple linear regression, we checked visually that the residuals were almost normally distributed. We also tested for multicollinearity with variance inflation factors. In the previous studies, a univariate test was conducted to examine the association between all variables added separately and an objective variable and *p*-values below 0.10 were considered for inclusion for adjustment in the multiple regression models [28,29]. To assess the changes in nutrient and food intake according to the DRIs-J score, tests for trend were used by modeling the median value of each quartile category as a continuous variable. Food groups included grains, potatoes, sugars, beans, nuts, vegetables, fruits, mushrooms, algae, fish and shellfish, meats, eggs, dairy products, fats and oils, confectioneries, beverages and seasoning, and spices, which are in line with the categories used in the Japanese National Health and Nutrition Survey. For almost all analyses, statistical significance was defined as a two-tailed *p*-value of <0.05. All statistical analyses were performed using SAS software (version 9.4; SAS Institute, Cary, NC, USA).

## 3. Results

Table 2 shows the total population, the number of deaths, and the mean SMR in 19 municipalities. Among the people in the Shiga prefecture, the mean all-cause SMR was 92.7 for men and 97.3 for women, during the years 2008 to 2012. The SMR of heart diseases was higher than that of other causes, especially in women. Compared with large cities, higher mortality rates were observed in some nonurbanized regions.

A total of 6057 individuals were included in the analysis from the 2015 HNSS. Table 3 shows the characteristics of the individuals by municipality. In terms of age, there were differences between cities, but not between the sexes. Overall, the energy intake of men was higher than that of women. Women’s health behaviors scores were higher than that of men; in particular, women were less likely to smoke and drink excessive alcohol compared with men.

Table 4 shows the association between the health behaviors score and all-cause and cause-specific SMR. The multiple models included appropriate variables selected using Spearman’s correlation. The coefficients having significant correlations between SMRs were followed. For men, all-cause SMR correlated with BMI (rho = −0.470, *p* = 0.042), and cancer SMR was correlated with age (rho = 0.520, *p* = 0.022) and energy intake (rho = 0.536, *p* = 0.018). SMR of heart diseases was correlated with age (rho = 0.416, *p* = 0.008) and BMI (rho = −0.396, *p* = 0.093). SMR of cerebrovascular diseases did not have a correlation with any variables. For women, all-cause SMR was correlated with BMI (rho = 0.569, *p* = 0.011), and cancer SMR was correlated with age (rho = −0.425, *p* = 0.070) and BMI (rho = −0.419, *p* = 0.074). SMR of heart diseases did not have any significant correlation, but SMR of cerebrovascular diseases was correlated with age (rho = 0.507, *p* = 0.027) and BMI (rho = 0.561, *p* = 0.012). In the multiple linear regression, the health behaviors score was not significantly associated with all-cause SMR for either sex. For cancer, there was a significant negative association between the health behaviors score and the cancer SMR only for women (*β* = −0.968, *p* = 0.011). Associations between the health behaviors score and SMR of heart diseases and cerebrovascular diseases were not found for either sex.

To examine which lifestyle factors of the health behaviors score were individually associated with a lower SMR of cancer in women, we conducted further analyses. Table 5 shows the relationship between five individual lifestyle factors of the health behaviors score and the cancer SMR. After adjusting for age and BMI, a greater proportion of individuals with better diet quality (*β* = −0.703, *p* = 0.048) and who never smoked (*β* = −0.780, *p* = 0.016) had a negative individual association with cancer SMR for women. These associations were not shown for the 3 other health behaviors. For men, there were no associations between cancer SMR and any individual health behaviors.

Despite the proportion of individuals who had higher diet quality, the DRIs-J score greater than or equal to the median score by sex in the study participants was not different between men and women; thus, why was there an association between diet quality and cancer SMR only in women? Table 6 and Table 7 shows the sex-specific changes in nutrients and foods intake according to quartile (Q) DRIs-J scores. Between both sexes, total DRIs-J scores did not differ (mean ± standard deviation was 3.3 ± 1.4, 6.5 ± 0.5, 9.4 ± 1.1, and 14.1 ± 1.8 in Q1, Q2, Q3, and Q4, respectively, in men, and 3.3 ± 1.4, 6.5 ± 0.5, 9.4 ± 1.1, and 14.3 ± 1.8 in Q1, Q2, Q3, and Q4, respectively, in women). Given energy intake increased significantly from Q1 to Q4, intake was represented per 1000 kcal. As shown in Table 6, almost all nutrient intake excluding fats, carbohydrates, sodium, and poly-unsaturated fatty acids increased as the DRIs-J score increased, regardless of sex. In women, however, intake of potassium, vitamin A, vitamin K, folate, and vitamin C were higher compared with men. In addition, the nutrient intake in women increased more from Q1 to Q4 compared with men, which is shown in the difference in mean values between Q1 and Q4. Potassium intake was 460 mg/1000 kcal/d in men, whereas it was 514 mg/1000 kcal/d in women. Vitamin A intake was 132 µgRAE/1000 kcal/d in men and 180 µgRAE/1000 kcal/d in women. For vitamin K, folate, and vitamin C, the intake was 70 µg/1000 kcal/d in men and 82 µg/1000 kcal/d in women, 67 µg/1000 kcal/d in men and 76 µg/1000 kcal/d in women, and 37 mg/1000 kcal/d in men and 45 mg/1000 kcal/d in women, respectively. In Table 7, the changes in food group intake according DRIs-J score were also similar between men and women. However, women’s fruit intake was particularly higher than that of men in all quartiles, and their intake increased more from Q1 to Q4 than that of men. The difference in the mean value of fruit intake between Q1 and Q4 was 51.9 g/1000 kcal/d in men and 65.4 g/1000 kcal/d in women.

## 4. Discussion

In the present study, it was found that the higher the health behaviors score (dietary adherence to DRIs-J, never smoking, nonexcessive alcohol drinking, regular exercise, and moderate sleep duration), the lower the cancer SMR in women. Regarding health behaviors alone, never smoking and higher dietary adherence to DRIs-J were individually associated with lower cancer SMR in women.

We found that a higher number of healthy behaviors were associated with a lower cancer SMR in women. Our investigation revealed that, of the health behaviors especially associated with cancer SMR, never smoking and higher diet quality were significantly associated with lower SMR of cancer among women. Previous studies have revealed associations between a reduced risk of cancer mortality and a healthy diet, particularly higher intake of fruits and vegetables and whole grains and lower intake of red and processed meat [30] and nonsmoking [31]. These findings were consistent with our results, which clarified that never smoking and high diet quality was particularly related to reduced cancer SMR in women. As for the association between sleep duration and a risk of cancer mortality, there are varied perspectives; however, the combination of a short sleep duration (<7 h/d) and less moderate to vigorous physical activity has been found to be synergistically associated with a higher risk of cancer mortality [32]. Our results suggest that combinations of these health behaviors were more strongly associated with a lower risk of cancer mortality than individual health behaviors in women.

Although the associations between a higher health behaviors score, higher diet quality, never smoking, and a risk of cancer SMR were not shown among men in our study, a prospective study of Korean men and women reported that the combined risk behaviors included never smoking, excessive alcohol drinking, less frequent physical activity, and inappropriate BMI were related to a higher risk of cancer mortality for both sexes [33]. Korean men had a high smoking rate of 29.2%, which is consistent with the smoking rate of men in our study, 30.1%, whereas this previous study did not sufficiently consider a dietary factor in scoring lifestyle factors due to the insufficient data on the nutrient survey. We suspect that in men, dietary factors influenced the risk of cancer mortality in relation to smoking. As for the dietary factors in our study, men consumed lower antioxidants; for example, vitamin A, folate, and vitamin C, in terms of foods, and less fruit compared with women. Although we could not consider the different smoking statuses because of the limited number of participants, a large-scale cross-sectional study in Japan had reported that smokers tended to consume fewer fruits and vegetables rich in antioxidants compared with nonsmokers [34]. In an investigation of more than 1000 adults, the plasma vitamin C concentration of smokers was lower than that of nonsmokers [35]. We suspect that the higher smoking rate and lower intake of antioxidants weakened the association between the health behavior score and cancer SMR among men in our study.

Other causes, such as all-cause, heart diseases, and cerebrovascular SMR, were not associated with the health behavior score in men or women. A prospective study of Japanese participants reported that a combination of health behaviors was associated with a lower risk of CVD, including stroke and coronary heart disease mortality [36]. The authors included BMI in the health behaviors, in addition to fruit and vegetable intake, smoking, alcohol intake, exercise, and sleeping. Another prospective study had reported a significant association between health behavior combinations and a risk of CVD mortality using a score including BMI [11]. The lack of an association between the health behaviors score and heart diseases and cerebrovascular SMR in our study appears to be explained partially by exclusion of the BMI component. A large population-based prospective study had shown an appropriate BMI was much more strongly related to a reduced risk of mortality from circulatory diseases compared with cancer [37]. Furthermore, high salt intake has been reported to be a risk factor for mortality, especially CVD mortality in Japanese women [38]. In the present study, crude salt equivalents increased in both sexes as DRIs-J score increased, although salt equivalents per 1000 kcal did not increase. This might partially weaken the relationship between the health behaviors score and the SMR of heart diseases and cerebrovascular diseases in our study.

This study has several strengths. The health behaviors score consisted of the factors based on significant evidence of an association with mortality risk. As far as we know, this study is one of a limited number of studies that has investigated the association between multiple lifestyle behaviors and a risk of cancer mortality. By the sex-specific analysis, the possibility was shown that the differences in lifestyle between sexes might be associated with a risk of cancer mortality. Our results will be useful for not only the Shiga prefecture but also Japan and other areas with similar lifestyle characteristics.

There are some limitations to the study. First, because it was an ecological study, our results cannot to be applied directly to individuals or reveal a causality between lifestyle factors and SMR. However, an ecological study is a useful method for a population approach that uses existing data. Second, the number of participants differed between municipalities. The numbers in some towns were particularly limited, however, so we used the SMR calculated from data spanning 5 years, which is considered to cover some yearly variations in the SMR in small population areas. Third, there were differences between the SMR survey years and lifestyle factors. Nevertheless, a previous study reported that the 1st to 7th causes of death in Japan did not change between 2005 and 2015 [1], so it was considered that the death profile between 2008 and 2012 in which we used the SMR did not change much. Fourthly, this study did not assess the association between sleep quality, which has shown its association with the risk of mortality from CVD [39]. However, as far as we know, few studies have examined the relationship between combined lifestyle factors, including sleep-related factors, and the risk of cause-specific death, especially cancers [32] Finally, information on lifestyle behaviors was obtained by self-reported questionnaires and the dietary record was conducted for only 1 day; thus, the dietary data might not reflect habitual intake. However, the nutrient intake of the individuals in our study was similar to the Japanese habitual nutrient intake [28].

## 5. Conclusions

This ecological study conducted in Shiga prefecture showed that a combination of healthy lifestyle factors, including never smoking, high adherence to a healthy diet, nonexcessive alcohol drinking, regular exercise, and moderate sleep duration were associated with a lower cancer SMR for women. In particular, not smoking and high dietary quality according to the DRIs-J were related to lower cancer SMR in women. In the future, it may be necessary to take measures that consider the differences in lifestyle between sexes.

## Figures and Tables

**Table 1:** Scoring of health behaviors on the study.

Health Behaviors	Scoring Method
Diet quality	1 = DRIs-J score ^1^ ≥ the median score by sex in the participants (the median score was 7.0 for both sexes).
Smoking	1 = never smoking.
Alcohol drinking	1 = consuming alcohol < 40 g/d for men or < 20 g/d for women.
Exercise behavior	1 = engaging in 30 min of exercise at least twice a week and continuing for a year.
Sleeping	1 = sleeping for ≥7 h/d and <9 h/d.
Total score	5

^1^ The score was constructed using the Dietary Reference Intakes for Japanese (DRIs-J), ranging from 0 to 19.

**Table 2:** Total population, all-cause and cause-specific number of deaths, and the mean SMR in 19 municipalities, 2008–2012, in the Shiga prefecture.

			All-Cause				Cancer				Heart Diseases				Cerebrovascular Diseases			
	Total Population ^1^		Death ^2^		SMR ^3^		Death ^2^		SMR ^3^		Death ^2^		SMR ^3^		Death ^2^		SMR ^3^	
Municipalities	Men	Women	Men	Women	Men	Women	Men	Women	Men	Women	Men	Women	Men	Women	Men	Women	Men	Women
Otsu	161,488	172,487	6749	6036	89.4	91.2	2343	1626	92.5	97.6	914	1081	86.4	92.9	621	631	87.5	85.0
Kusatsu	66,754	62,100	2022	1795	85.4	94.3	736	496	91.3	98.9	265	332	81.2	101.9	160	176	74.0	83.9
Moriyama	37,361	38,591	1285	1164	84.6	97.3	442	300	86.1	96.1	171	199	81.0	96.9	98	105	69.3	79.6
Ritto	31,332	31,509	923	872	90.4	108	336	200	96.6	91.6	137	176	97.6	130.0	90	81	96.8	92.5
Yasu	24,596	24,922	999	937	92.3	103.5	350	228	95.1	96.9	133	174	88.3	110.4	74	83	73.5	81.8
Koga	44,837	45,625	2225	2118	98.4	101.6	750	469	99.4	92.3	357	411	112.5	109.8	174	228	81.3	95.8
Konan	27,200	25,502	996	816	99.3	96.9	356	223	102.2	102.7	214	278	154.7	191.1	82	75	89.5	80.4
Omihachiman	39,511	41,024	1810	1709	95.3	101.9	633	410	97.9	96.6	277	298	104.6	100.8	140	154	78.7	81.4
Higashiomi	55,700	57,436	2592	2559	91.9	92.7	838	555	91.4	86.5	347	506	86.9	100.7	237	302	88.6	95.2
Hino	11,185	11,304	603	637	94.7	104.4	180	117	87.9	81.3	96	153	105.9	137.1	60	103	98.7	145.4
Ryuo	6789	6046	265	312	95.3	120.8	89	68	95.0	107.1	23	58	59.7	125.6	35	29	134.8	98.2
Hikone	54,432	56,088	2347	2228	92.3	98.6	811	509	96.3	89.7	352	523	98.6	130.6	210	270	87.5	105.3
Aiso	9626	9872	455	433	99.1	101.4	136	99	90.7	97.7	82	100	126.8	129.6	39	46	89.4	94.0
Toyosato	3582	3890	190	207	98.6	86.6	51	60	79.8	115.7	26	39	95.8	87.4	23	20	124.9	71.7
Kora	3556	3897	230	174	118.9	98.6	74	44	112.0	94.9	40	37	147.9	118.8	22	21	120.2	104.4
Taga	3665	4081	244	291	97.0	98.4	81	41	99.8	65.8	35	61	97.2	109.7	29	40	118.9	115.7
Nagahama	59,684	61,771	3175	3107	96.1	99.2	1011	678	93.3	92.4	465	580	99.6	101.5	301	374	95.3	103.6
Maibara	19,267	20,337	1164	1133	101.7	101.8	364	237	97.9	92.6	178	241	109.6	117.8	111	133	100.7	103.1
Takashima	25,365	26,627	1531	1520	93.6	100.2	517	401	96.0	113.1	197	266	85.0	96.0	147	165	93.6	94.3

SMR: standardized mortality ratio, ^1^ from the national census in 2009, ^2^ total of deaths, 2008–2012, ^3^ mean of SMR, 2008–2012.

**Table 3:** Participants’ characteristics in 19 municipalities in the 2015 Shiga Health and Nutrition Survey.

	n		Age (Years)		BMI (kg/m^2^)		Energy Intake (kcal/d)		Health Behaviors Score ^1^ (Points)		Health Behaviors									
											Diet Quality Score = 1 (%)		Smoking Score = 1 (%)		Alcohol Drinking Score = 1 (%)		Exercise Behavior Score = 1 (%)		Sleeping Score = 1 (%)	
Municipalities	Men	Women	Men	Women	Men	Women	Men	Women	Men	Women	Men	Women	Men	Women	Men	Women	Men	Women	Men	Women
Otsu	650	814	58.1	57.2	23.3	21.9	1960	1701	2.5	3.1	63.2	64.9	32.6	92.5	90.2	95.8	37.2	34.2	28.3	20.4
Kusatsu	166	205	50.1	47.7	23.4	21.2	1845	1670	2.2	2.7	44.6	51.2	42.8	84.9	90.4	91.2	27.7	22.0	13.3	22.0
Moriyama	143	168	50.2	49.3	23.0	21.4	1798	1555	2.2	2.8	53.8	54.2	25.2	85.1	86.7	94.0	29.4	26.8	21.7	22.0
Ritto	117	145	58.6	57.9	23.5	22.4	1996	1685	2.5	3.1	67.5	63.4	29.9	90.3	94.9	97.2	29.1	29.0	26.5	27.6
Yasu	81	86	53.1	51.0	23.4	21.6	1896	1571	2.2	2.6	48.1	44.2	28.4	91.9	93.8	91.9	25.9	14.0	23.5	18.6
Koga	244	284	56.3	57.8	23.2	22.1	1970	1649	2.3	3.0	61.1	65.1	26.2	91.2	87.7	96.1	25.4	26.4	27.0	23.2
Konan	148	174	55.1	54.8	22.5	21.4	1957	1614	2.4	2.9	56.8	56.3	32.4	91.4	93.2	94.3	33.1	29.9	25.7	17.8
Omihachiman	171	181	56.6	55.8	23.3	22.4	1947	1670	2.5	3.0	62.0	66.3	26.9	91.7	89.5	95.0	39.2	29.3	27.5	20.4
Higashiomi	208	265	56.8	58.0	23.6	22.1	2012	1703	2.4	3.0	66.3	67.2	28.8	92.8	92.8	98.9	27.4	28.3	28.8	17.0
Hino	57	58	55.7	57.7	22.5	22.5	1905	1659	2.2	3.1	64.9	67.2	31.6	93.1	84.2	98.3	19.3	20.7	17.5	27.6
Ryuo	27	30	50.3	57.3	23.1	22.5	2017	1741	2.2	2.8	55.6	53.3	29.6	86.7	92.6	90.0	18.5	20.0	25.9	30.0
Hikone	168	192	53.8	53.0	23.1	21.7	1884	1570	2.1	2.7	51.8	46.9	25.0	84.9	87.5	94.8	22.0	18.2	21.4	25.5
Aiso	34	40	50.8	51.8	23.2	21.5	1906	1523	2.2	2.8	47.1	50.0	32.4	87.5	94.1	100.0	26.5	17.5	17.6	22.5
Toyosato	16	26	48.1	45.7	21.5	21.8	1807	1681	1.7	2.4	43.8	38.5	25.0	76.9	81.3	100.0	6.3	3.8	12.5	23.1
Kora	22	19	59.9	59.9	22.5	22.1	2077	1668	2.4	3.2	86.4	68.4	13.6	100.0	95.5	100.0	27.3	21.1	18.2	31.6
Taga	26	28	52.7	55.9	23.5	22.5	2147	1714	2.3	3.0	73.1	64.3	23.1	92.9	80.8	96.4	30.8	21.4	23.1	28.6
Nagahama	310	334	50.6	51.7	23.4	21.8	1893	1570	2.1	2.7	48.4	45.8	28.7	89.5	86.8	96.4	19.7	18.3	28.4	23.7
Maibara	89	101	55.6	56.3	22.7	22.2	1874	1628	2.3	3.0	59.6	51.5	30.3	95.0	88.8	98.0	20.2	26.7	27.0	26.7
Takashima	113	117	54.7	56.8	23.5	22.1	1887	1619	2.1	2.7	49.6	48.7	31.9	82.9	83.2	95.7	20.4	20.5	27.4	18.8

The data of age, BMI, energy intake, and the health behaviors score indicated the mean values. ^1^ Consisted of 5 health behaviors listed on the right, ranging from 0 to 5. Details of scoring are shown in Table 1.

**Table 4:** The associations between the health behaviors score and SMRs of all-cause and cause-specific in multiple linear regression analysis.

	Men		Women	
Cause	*β*	*p* Value ^1^	*β*	*p* Value ^1^
All-cause	0.379	0.138	−0.126	0.621
Cancer	0.043	0.886	−0.968	0.011
Heart diseases	0.345	0.376	0.174	0.477
Cerebrovascular diseases	−0.054	0.828	0.178	0.631

*β*: standardized beta coefficient, SMR: standardized mortality ratio, ^1^*p* value was from the multiple liner regression analysis between the health behaviors score and SMRs. The details of scorings of health behaviors are shown in Table 1. Adjusted for variables with *p* < 0.10 when investigated Spearman’s correlation coefficients between SMRs of each cause and age (continuous), BMI (continuous), energy intakes (continuous), and subjective health perception (category) by sex. *p* < 0.05 was significant.

**Table 5:** The associations between individual lifestyle factors of the health behaviors score and cancer SMR.

	Men		Women	
Item	*β*	*p* Value ^1^	*β*	*p* Value ^1^
Diet quality	−0.119	0.759	−0.703	0.048
Smoking	−0.138	0.469	−0.780	0.016
Alcohol drinking	0.190	0.307	−0.097	0.696
Regular exercise	0.075	0.714	−0.298	0.380
Sleep duration	0.010	0.958	−0.210	0.466

*β*: standardized beta coefficient, SMR: standardized mortality ratio, ^1^*p* value was from the multiple liner regression analysis between the health behaviors score and SMRs. The details of scorings of health behaviors are shown in Table 1. Adjusted for age (continuous), energy intake (continuous) for men, age (continuous), BMI (continuous) for women with *p* < 0.10 when investigated Spearman’s correlation coefficients between cancer SMR and age, BMI, energy intakes, and subjective health perception by sex. *p* < 0.05 was significant.

**Table 6:** Changes in energy and nutrient intake per 1000 kcal according to the DRIs-J scores.

	Men					Women				
	DRIs-J Score ^1^					DRIs-J Score ^1^				
	Q1	Q2	Q3	Q4		Q1	Q2	Q3	Q4	
	n = 924	n = 485	n = 823	n = 558	*p* for Trend	n = 1078	n = 562	n = 843	n = 784	*p* for Trend
Energy (kcal/d)	1542 ± 368	1871 ± 354	2097 ± 369	2382 ± 332	<0.001	1321 ± 275	1577 ± 305	1765 ± 329	2021 ± 375	<0.001
Nutrient (/1000 kcal/d)										
Protein (g)	36.4 ± 8.4	38.5 ± 8.3	39.6 ± 7.6	40.2 ± 6.8	<0.001	37.1 ± 8.1	39.0 ± 7.9	40.1 ± 7.7	41.2 ± 7.1	<0.001
Animal protein (g)	20.0 ± 9.0	21.4 ± 8.8	22.7 ± 8.1	22.1 ± 6.9	<0.001	19.5 ± 8.8	21.3 ± 8.5	22.3 ± 8.3	22.0 ± 7.3	<0.001
Plant protein (g)	16.5 ± 4.4	17.1 ± 4.2	16.9 ± 3.9	18.2 ± 4.0	<0.001	17.6 ± 4.4	17.7 ± 4.1	17.9 ± 4.0	19.2 ± 4.2	<0.001
Fat (g)	29.8 ± 9.4	29.9 ± 8.0	29.7 ± 7.6	29.4 ± 6.6	0.713	31.5 ± 9.5	31.0 ± 8.2	31.0 ± 7.6	30.6 ± 6.7	0.090
Carbohydrate (g)	135.9 ± 26.1	133.6 ± 21.4	133.5 ± 20.3	135.2 ± 18.7	0.080	137.1 ± 25.1	136.4 ± 21.1	136.1 ± 20.8	137.8 ± 18.0	0.412
Sodium (mg)	2165 ± 896	2278 ± 936	2193 ± 804	2189 ± 745	0.124	2278 ± 933	2242 ± 783	2246 ± 853	2300 ± 777	0.510
Potassium (mg)	1049 ± 314	1158 ± 320	1266 ± 337	1509 ± 354	<0.001	1157 ± 332	1286 ± 364	1386 ± 364	1671 ± 396	<0.001
Calcium (mg)	213 ± 101	250 ± 118	258 ± 101	325 ± 124	<0.001	257 ± 117	280 ± 121	302 ± 118	373 ± 139	<0.001
Magnesium (mg)	115 ± 31	127 ± 33	135 ± 34	158 ± 38	<0.001	125 ± 33	135 ± 35	144 ± 35	169 ± 41	<0.001
Phosphorus (mg)	483 ± 113	525 ± 115	543 ± 106	585 ± 115	<0.001	512 ± 116	548 ± 123	572 ± 114	621 ± 118	<0.001
Iron (mg)	3.6 ± 1.1	4.1 ± 1.3	4.3 ± 1.2	4.9 ± 1.3	<0.001	4.0 ± 1.4	4.3 ± 1.3	4.5 ± 1.2	5.4 ± 1.6	<0.001
Zinc (mg)	4.3 ± 1.0	4.4 ± 1.0	4.5 ± 0.9	4.6 ± 1.0	<0.001	4.3 ± 0.9	4.4 ± 1.0	4.5 ± 0.8	4.7 ± 0.9	<0.001
Copper (mg)	0.56 ± 0.12	0.59 ± 0.15	0.61 ± 0.13	0.67 ± 0.14	<0.001	0.57 ± 0.12	0.61 ± 0.14	0.64 ± 0.13	0.70 ± 0.14	<0.001
Vitamin A (μgRAE)	243 ± 188	253 ± 245	324 ± 399	375 ± 266	<0.001	269 ± 196	305 ± 321	337 ± 253	449 ± 515	<0.001
Vitamin D (μg)	3.3 ± 4.3	4.3 ± 4.5	4.7 ± 5.4	5.1 ± 4.6	<0.001	3.3 ± 4.8	4.8 ± 6.5	4.8 ± 4.9	5.6 ± 5.0	<0.001
Vitamin E (mg)	3.2 ± 1.3	3.5 ± 1.4	3.7 ± 1.5	4.2 ± 1.6	<0.001	3.5 ± 1.4	3.8 ± 1.6	4.0 ± 1.6	4.6 ± 1.7	<0.001
Vitamin K (μg)	113 ± 89	127 ± 93	141 ± 96	183 ± 122	<0.001	128 ± 103	141 ± 105	159 ± 109	210 ± 128	<0.001
Vitamin B_1_ (mg)	0.45 ± 0.21	0.49 ± 0.23	0.50 ± 0.22	0.53 ± 0.20	<0.001	0.46 ± 0.19	0.48 ± 0.21	0.50 ± 0.20	0.54 ± 0.17	<0.001
Vitamin B_2_ (mg)	0.51 ± 0.21	0.57 ± 0.24	0.61 ± 0.22	0.66 ± 0.20	<0.001	0.57 ± 0.28	0.61 ± 0.24	0.66 ± 0.22	0.72 ± 0.21	<0.001
Niacin (mgNE)	7.5 ± 3.1	8.5 ± 4.0	9.1 ± 3.7	9.2 ± 2.8	<0.001	7.5 ± 3.0	8.4 ± 3.2	8.7 ± 3.4	9.3 ± 3.0	<0.001
Vitamin B_6_ (mg)	0.56 ± 0.18	0.60 ± 0.19	0.67 ± 0.19	0.74 ± 0.17	<0.001	0.57 ± 0.18	0.62 ± 0.18	0.68 ± 0.19	0.77 ± 0.18	<0.001
Vitamin B_12_ (mg)	2.6 ± 3.6	3.5 ± 3.5	4.1 ± 4.3	4.2 ± 3.9	<0.001	2.5 ± 3.2	3.5 ± 3.7	3.7 ± 3.7	4.5 ± 4.2	<0.001
Folate (μg)	126 ± 51	145 ± 62	160 ± 63	193 ± 65	<0.001	142 ± 56	158 ± 61	174 ± 63	218 ± 83	<0.001
Pantothenic acid (mg)	2.65 ± 0.63	2.80 ± 0.64	2.97 ± 0.67	3.19 ± 0.65	<0.001	2.79 ± 0.66	2.94 ± 0.66	3.17 ± 0.68	3.46 ± 0.75	<0.001
Vitamin C (mg)	37 ± 28	43 ± 34	54 ± 35	74 ± 39	<0.001	44 ± 34	54 ± 38	63 ± 39	89 ± 45	<0.001
Saturated fatty acid (g)	7.86 ± 3.10	7.81 ± 2.79	7.75 ± 2.70	7.48 ± 2.34	0.081	8.64 ± 3.23	8.47 ± 3.18	8.42 ± 2.88	8.14 ± 2.65	0.005
Fatty acid, mono-unsaturated (g)	10.59 ± 4.01	10.39 ± 3.39	10.34 ± 3.29	9.89 ± 2.98	0.003	10.96 ± 4.12	10.55 ± 3.40	10.52 ± 3.28	10.07 ± 2.97	<0.001
Fatty acid, poly-unsaturated (g)	6.52 ± 2.65	6.72 ± 2.38	6.60 ± 2.21	6.84 ± 2.10	0.066	6.76 ± 2.81	6.79 ± 2.43	6.81 ± 2.31	7.04 ± 2.29	0.082
Cholesterol (mg)	173.9 ± 100.6	179.5 ± 91.8	192.2 ± 99.0	181.9 ± 83.9	0.001	178.2 ± 108.9	180.7 ± 95.6	199.8 ± 98.2	184.0 ± 87.8	<0.001
Dietary fiber (g)	6.8 ± 2.4	7.4 ± 2.6	8.0 ± 2.7	10.0 ± 3.3	<0.001	7.6 ± 2.6	8.1 ± 2.7	8.9 ± 3.0	11.1 ± 3.4	<0.001

Q: quartile, values indicate mean ± standard deviation. ^1^ The score was constructed using the Dietary Reference Intakes for Japanese (DRIs-J), ranging from 0 to 19. *p* for trend < 0.05 was significant.

**Table 7:** Changes in food group intake per 1000 kcal according to the DRIs-J scores.

	Men					Women				
	DRIs-J Score ^1^					DRIs-J Score ^1^				
	Q1	Q2	Q3	Q4		Q1	Q2	Q3	Q4	
	n = 924	n = 485	n = 823	n = 558	*p* for Trend	n = 1078	n = 562	n = 843	n = 784	*p* for Trend
Food intake (g/1000 kcal/d)										
Grains	265.6 ± 85.5	246.4 ± 74.6	230.3 ± 65.6	206.7 ± 58.7	<0.001	246.4 ± 82.5	226.8 ± 74.4	211.4 ± 67.5	183.5 ± 55.9	<0.001
Potatoes	28.1 ± 34.8	29.5 ± 33.9	31.3 ± 38.4	34.7 ± 36.0	<0.001	32.5 ± 37.8	34.6 ± 40.8	34.2 ± 40.2	36.9 ± 41.3	0.141
Sugars	2.7 ± 3.5	3.0 ± 3.5	3.4 ± 3.9	3.6 ± 3.7	<0.001	3.2 ± 4.2	3.8 ± 4.0	3.6 ± 4.2	4.2 ± 4.4	<0.001
Beans	22.6 ± 34.2	29.9 ± 37.6	30.3 ± 34.3	39.9 ± 38.0	<0.001	27.7 ± 39.2	32.0 ± 39.7	36.1 ± 41.0	46.1 ± 41.9	<0.001
Nuts	0.8 ± 2.5	0.8 ± 2.7	1.3 ± 3.2	2.0 ± 4.7	<0.001	1.0 ± 2.9	1.1 ± 2.9	1.4 ± 3.2	2.3 ± 5.2	<0.001
Vegetables	144.0 ± 87.1	158.8 ± 96.5	168.9 ± 84.0	203.8 ± 99.6	<0.001	156.1 ± 93.1	166.9 ± 91.9	179.0 ± 98.3	216.9 ± 105.7	<0.001
Yellow and green vegetables	47.5 ± 43.9	48.7 ± 41.5	60.9 ± 47.9	81.7 ± 57.3	<0.001	52.6 ± 49.4	59.2 ± 50.8	67.8 ± 53.8	91.8 ± 62.9	<0.001
Other vegetables	89.1 ± 66.8	100.3 ± 70.4	97.6 ± 63.4	110.2 ± 73.1	<0.001	95.9 ± 69.3	98.9 ± 65.1	99.8 ± 72.0	111.4 ± 77.3	<0.001
Fruits	26.6 ± 50.3	32.1 ± 53.0	51.0 ± 61.9	78.5 ± 67.3	<0.001	38.0 ± 60.9	54.2 ± 67.8	71.6 ± 73.2	103.4 ± 76.4	<0.001
Mushrooms	6.9 ± 14.8	8.8 ± 18.9	9.0 ± 17.3	11.1 ± 18.7	<0.001	7.1 ± 13.4	8.5 ± 14.9	9.8 ± 15.7	11.3 ± 16.6	<0.001
Algae	4.5 ± 11.7	5.2 ± 11.6	5.7 ± 11.2	6.9 ± 10.8	0.001	5.0 ± 14.6	6.2 ± 14.7	5.3 ± 10.6	7.4 ± 14.0	0.001
Fish and shellfish	31.9 ± 37.2	42.0 ± 38.7	46.2 ± 40.5	45.8 ± 35.1	<0.001	30.2 ± 36.2	42.8 ± 39.4	43.0 ± 38.6	48.3 ± 36.9	<0.001
Meats	61.3 ± 43.8	56.8 ± 44.0	58.0 ± 42.6	50.3 ± 36.5	<0.001	54.9 ± 40.5	50.5 ± 38.8	50.4 ± 39.7	42.7 ± 31.4	<0.001
Eggs	21.4 ± 21.8	21.0 ± 19.0	22.6 ± 21.2	20.4 ± 16.9	0.254	22.4 ± 24.3	20.5 ± 20.2	25.0 ± 20.8	20.4 ± 17.8	<0.001
Dairy products	35.9 ± 59.7	36.7 ± 53.5	39.1 ± 53.0	51.8 ± 51.5	<0.001	54.0 ± 77.0	56.8 ± 69.1	64.1 ± 69.0	76.5 ± 69.5	<0.001
Fat and oil	6.1 ± 5.5	5.2 ± 4.4	5.0 ± 4.2	4.4 ± 3.9	0.009	5.8 ± 5.6	5.3 ± 4.8	4.9 ± 4.4	4.5 ± 4.1	<0.001
Confectioneries	7.7 ± 19.8	8.8 ± 17.3	9.6 ± 18.3	11.8 ± 17.5	<0.001	13.6 ± 26.7	15.4 ± 24.5	17.8 ± 25.2	19.5 ± 23.8	<0.001
Beverages	195.5 ± 233.1	224.2 ± 223.8	242.5 ± 234.8	236.0 ± 207.3	<0.001	208.5 ± 231.8	233.2 ± 240.4	206.5 ± 204.5	242.7 ± 235.6	0.002
Seasoning and spices	67.7 ± 77.4	66.0 ± 63.9	65.7 ± 65.9	63.5 ± 60.3	<0.001	68.5 ± 81.8	66.8 ± 68.9	67.0 ± 68.4	69.1 ± 72.4	0.911

Q: quartile, values indicate mean ± standard deviation. ^1^ The score was constructed using the Dietary Reference Intakes for Japanese (DRIs-J), ranging from 0 to 19. *p* for trend < 0.05 was significant.
